# Experimental study on the gas desorption law in coal affected by dynamic water injection

**DOI:** 10.1038/s41598-023-49607-y

**Published:** 2023-12-16

**Authors:** Tianwei Shi, Aiwen Wang, Lianpeng Dai, Gang Wang

**Affiliations:** Institute of Disaster Rock Mechanics, Liaoning University, Shenyang, 110036 China

**Keywords:** Energy science and technology, Engineering

## Abstract

Water from hydraulic technology affects the desorption of gas from coal seams. Gas desorption behavior is critical information for gas control in coal mines. In this study, a designed coal seam water injection simulation experimental device was utilized to conduct dynamic water injection experiments on coal samples at different adsorption equilibrium pressures, analyzing the gas desorption law under dynamic water injection, as well as the role of water replacement, water imbibition and water blockage in gas desorption. The results showed that water altered the gas desorption rate in coal, causing fluctuating attenuation of the desorption rate of a water-injected coal sample (WCS). Under the same adsorption equilibrium pressure, the relationship between the desorption rate of the WCS and the non-water-injected coal samples (NCS) underwent a transition in desorption time. In contrast to the NCS desorption curves, the WCS desorption curves lacked a rapid growth phase and exhibited only a slow growth phase and a stopping phase. Water imbibition and water replacement promoted the desorption of gas in the non-wet area during the water injection process, while it inhibited the desorption of gas in the wet area. Under the effects of water imbibition, water blockage, and water replacement, the discharge rate of WCS is greater than the desorption rate of NCS, indicating that water injection increases the total amount of gas desorption. The study results have significant implications for gas extraction and the prevention and control of coal and gas outbursts.

## Introduction

Gas is a type of clean energy with a high combustion value, and it is a hazard in coal mines. Over 70% of the coal seams in China are characterized by a high gas content and low permeability, which makes it difficult to extract gas for effective utilization^1^. When deep mining begins in China's major mining areas, the gas content in the coal seams increases, intensifying the associated risks and hazards caused by gas-related incidents^2–4^. To reduce the impact of gas on mine safety, a combination of hydraulic technology and gas extraction is often adopted as a control measure^5,6^. Hydraulic technology can improve the permeability of coal seams and enhance the effect of gas extraction from coal seams^7,8^. However, the water used in hydraulic technology has a great impact on the desorption of gas, which is directly related to gas extraction and gas-related disasters^9–11^. Therefore, it is important to study the influence of water on gas desorption from coal seams.

According to the time sequence of water and gas entering the coal body, water entry can be divided into two types, defined here as “early water intrusion” and “late water intrusion”. In early water intrusion, water enters the coal body before gas; that is, the coal is first wetted by water and then adsorbs gas. In late water intrusion, gas enters the coal body before water; that is, the coal first absorbs gas and then is immersed in water. Studies of gas desorption related to early water intrusion have reported that water enters the pores and fissures of coal samples and blocks the gas desorption paths, reducing the gas desorption rate and amount^12–17^. The adsorption capacity of water is stronger than that of methane^18,19^. Water occupies adsorption sites before gas does, resulting in a reduction in the gas adsorption amount. Therefore, in the case of early water intrusion, the higher the moisture content of the coal is, the smaller the gas desorption amount, and the slower the desorption rate. This situation is different from that when using hydraulic technology in a coal seam. In the field, the coal seam initially contains gas, and the water used in the hydraulic technique soaks into the coal seam afterward, which corresponds to late water intrusion. Zhang et al.^20^ studied the influence of late water intrusion on gas desorption characteristics. Their experimental results showed that water displaces gas. When the moisture content of coal increased from 0 to 1.8%, the total gas desorption amount of the coal samples decreased by 26.65%, and the initial diffusion coefficient decreased by 38%. Li et al.^21^ found that the displacement effect of late water intrusion increased the total gas desorption from coal samples compared to that of dry coal samples. Wu et al.^22^ found that at first, water inhibited the gas desorption rate from coal samples, but after a long desorption time, the displacement effect increased the gas desorption amount. Ni et al.^23^ found that static pressure fracturing and low pressure-low frequency pulsating fracturing inhibited gas desorption, while low pressure-high frequency, high pressure-low frequency and high pressure-high frequency pulsating fracturing promoted gas desorption from coal samples. Xiao et al.^24^ experimentally investigated the impact of late water intrusion on gas desorption and found that although displacement occurred, water still inhibited the desorption rate and desorption amount. In studies of the influence of late water intrusion on gas desorption, water reduced the gas desorption rate, but when the displacement effect of water is considered, there are two completely opposite conclusions about the influence of water on the gas desorption amount. In studies of late water intrusion, water is generally injected into the adsorption tank after the adsorption equilibrium pressure is stable while the exhaust port is closed, and following the gas readjustment inside the adsorption tank, the exhaust port is opened to allow depressurization and desorption^24–30^. During the injection of water, the exhaust port is closed, and the gas fluidity is limited. In fact, during the process of water intrusion into a coal seam, free gas can flow forward along the pores and fissures of the coal seam^31^. Additionally, after hydraulic measures are implemented, water retained within the coal seam undergoes capillary action leading to water imbibition^32,33^. The water imbibition can induce displacement and adsorption of gas. Indeed, the existing experiments on water intrusion lack the simulation of the dynamic process of gas displacement by water within coal seams, and fail to consider the combined effects of retained water imbibition and displacement on gas desorption.

Based on the above analysis, this study first conducted an experiment where water replaced gas to study the displacement effect of water on gas. Second, a newly designed water injection device was utilized to experimentally simulate dynamic water injection and investigate its impact on gas desorption from coal samples. Moreover, the effects of water sealing, displacement, and imbibition on gas desorption were also explored. The study results have significant implications for gas extraction and the prevention and control of coal and gas outbursts.

## Methods

### Preparation of coal samples

Coal samples were taken from the Pingmei No. 11 mine, Pingdingshan City, Henan Province. The coal seam mined in Pingmei No. 11 mine is identified as an outburst coal seam. As the coal used in this study is soft, a briquette (size of approximately ø 50 × 100 mm) was used in the experiment to study the dynamic water displacement of gas. The collected coal blocks were crushed, and standard sieves were used to screen out pulverized coal with particle sizes of 1–3 mm and 0.25–0.38 mm. Water was added as a binder to pulverized coal with a particle size of 0.25–0.38 mm, after which the sample was mixed evenly and put into a briquette-making device. The device was pressurized to 100 MPa on a hydraulic testing machine and unloaded after 30 min of steady pressure. The briquettes and pulverized coal with a particle size of 1–3 mm were placed in a drying oven at 60 °C for 24 h^34^. The pulverized coal with a particle size of 1–3 mm was used to study the replacement of adsorbed gas by water.

### Experimental device

#### Experimental device for studying the replacement of gas by water

**Figure 1:**
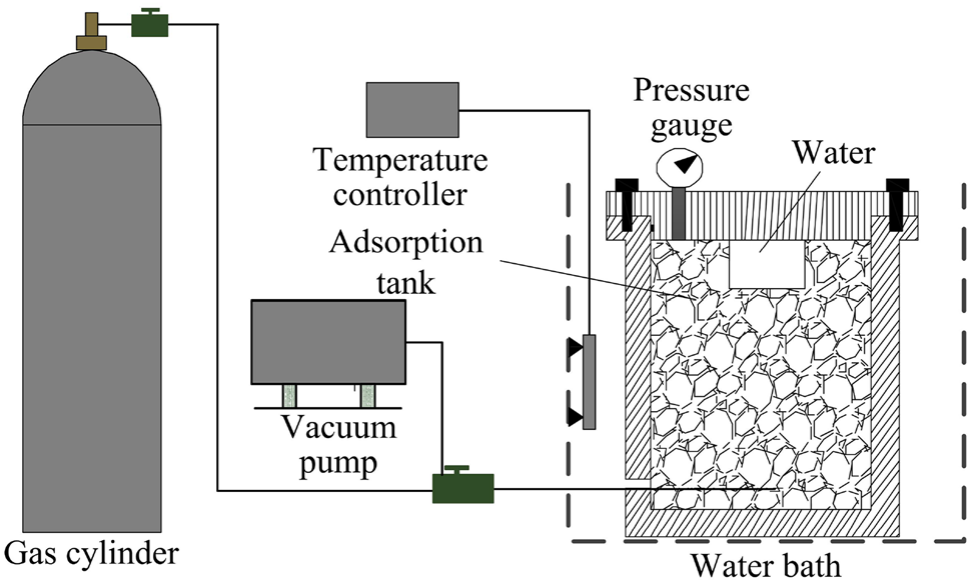
Experimental device for studying the replacement of adsorbed gas by water.

In order to explain the law of the effect of water on gas desorption, the phenomenon of water replacement of adsorbed gas was first explored. As shown in Fig. 1, the experimental device for studying the replacement of gas by water included an adsorption tank with a built-in water storage tank, water bath, vacuum pump, and other components.

#### Experimental device for the simulation of water injection

**Figure 2:**
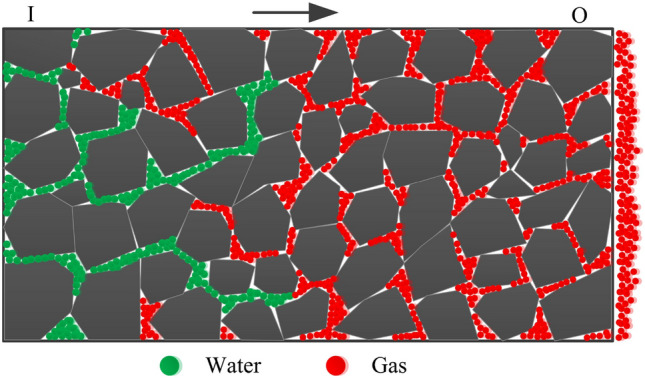
Water flow in coal seams.

The use of hydraulic technology to inject water into coal seams is taken as an example. As shown in Fig. 2, water flows from end I to end O along the flow direction during water injection, and gas is driven out from end O along the flow direction. Therefore, when using hydraulic technology, dynamic water flow can wet the coal while displacing gas.

**Figure 3:**
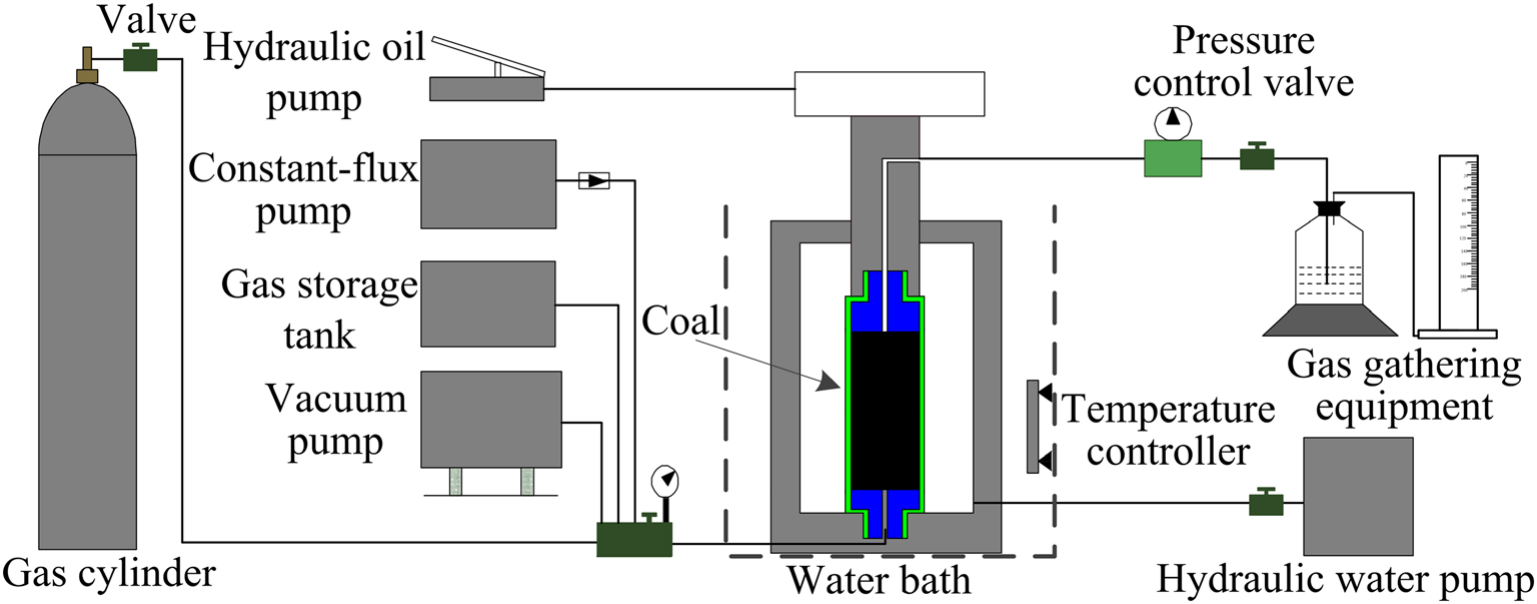
Water injection simulation experimental device.

According to the above analysis, an experimental device was designed to simulate the dynamic water displacement of gas as shown in Fig. 3. The device was composed of an adsorption tank, vacuum pump, temperature controller, constant-flux pump, axial and confining pressure loading equipment, gas collection equipment, and other components. The constant-flux pump connected to the water inlet at the air inlet end of the adsorption tank controlled the water injection pressure. The maximum water injection pressure and maximum water injection flow of the constant-flux pump were 40 MPa and 10 mL/min, respectively. The pressure control valve connected to the discharge end at the exhaust end of the adsorption tank controlled the pressure at the discharge end. The pressure control valve regulated the pressure in the range of 0–2.5 MPa. The axial pressure and surrounding pressure in the experiment were controlled by the hydraulic oil pump and hydraulic water pump, respectively.

### Experimental procedure

#### Experiment of the displacement of gas by water

A total of 400 g of pulverized coal with a particle size of 1.0–3.0 mm was placed into the adsorption tank. The water storage tank was placed on the pulverized coal. The weight of water in the tank was 40 g. After the equipment was assembled, the outlet of the water storage tank was closely connected to the cover of the adsorption tank, and a waterproof cloth was placed between the tanks to reduce the effect of water evaporation on the adsorption equilibrium. High-pressure helium was injected into the experimental device to check the integrity of the tank. Then, the vacuum pump was connected for degassing until the vacuum level was less than 10 Pa. The gas cylinder was connected, 99.99% pure methane was injected, and adsorption stopped when the change in pressure in the adsorption tank was less than 0.01 MPa after 2 h. The adsorption tank was repeatedly inverted to cause the water in the water storage tank to flow out and wet the pulverized coal. The gas pressure in the adsorption tank was recorded at intervals.

#### Dynamic water injection simulation experiment

Desorption experiments of anhydrous coal samples and dynamic water-injected coal samples under different adsorption equilibrium pressures were performed in this study. The injection pressure of dynamic water in the experiment was 4 MPa. The pressure at the discharge end of the coal sample was equal to the adsorption equilibrium pressure of the coal sample so that the gas discharged during water injection was caused by water. The experimental steps were as follows.

1. Check for airtightness. The axial and confining pressures of the coal sample were loaded to 8 MPa. High-pressure helium was injected to check the airtightness of the experimental device. And the pipeline volume was measured at both ends of the adsorption tank.
2. Gas adsorption. After vacuum degassing, the gas storage tank and the adsorption tank were connected to start gas adsorption. The temperature of the adsorption tank was set to 20 °C, and adsorption time was about 6 h.
3. Gas desorption. Start the desorption experiment by opening the exhaust end until the exhaust volume at the exhaust end is less than 0.1 mL/min.
4. Water injection. Step (2) was repeated, and water injection was started until there was water discharged from the exhaust end before stopping. Then, the desorption experiment was started.

The amount of gas obtained from the experiment was converted to the amount of gas in the standard state.

## Results

### Replacement effect

**Figure 4:**
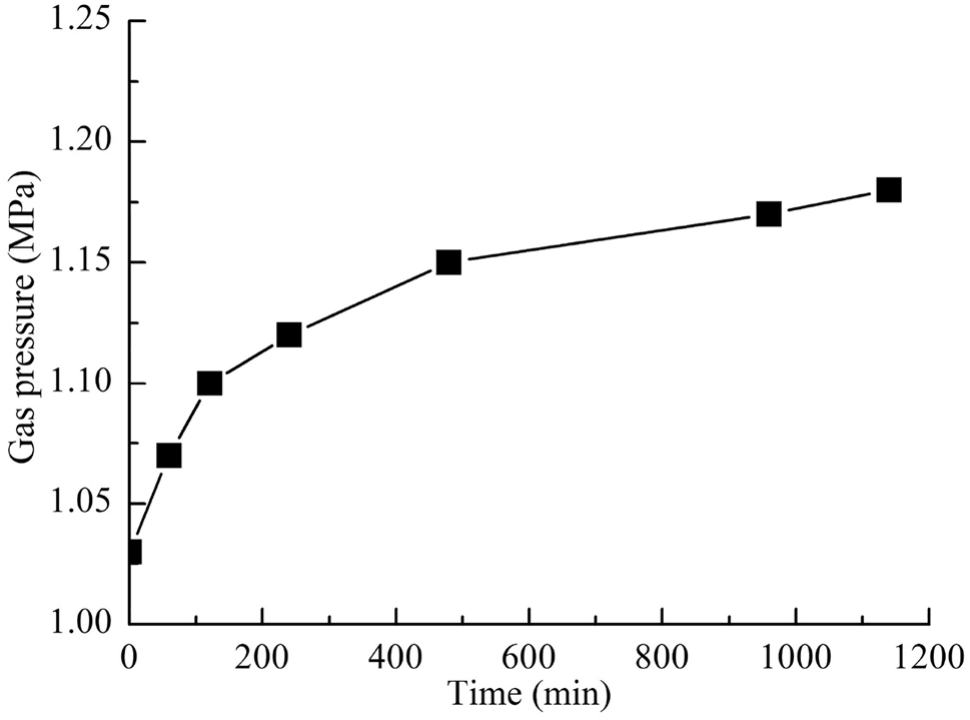
Gas pressure change after pulverized coal wetting.

As shown in Fig. 4, after the pulverized coal was wetted with water, the methane pressure in the adsorption tank gradually increased, but the rate gradually decreased. Since a built-in water storage tank was used, the volume of free space in the adsorption tank did not change during the whole process of wetting the pulverized coal. Therefore, the increase in gas pressure in the adsorption tank was caused solely by the increase in gas volume, which could only have come from adsorbed methane. Therefore, the experiment indicated that water replaced methane in the adsorption tank.

After the contact of water with pulverized coal, the surface tension made the water spread gradually in the pulverized coal. And the expansion of areas wetted by water in the pulverized coal led to the increase of replacement areas. The stronger adsorption force of coal to water made the adsorption sites of coal occupied by water, and a large amount of adsorbed methane in coal converted into free gas. With the weakening of water diffusion and the decrease of adsorbed methane in the water wetted area, the displacement amount of methane decreased. The rate of increase of gas pressure in the adsorption tank gradually decreased. This showed that the amount of gas replacement was related to the adsorption amount of methane.

### Effect of water injection on the moisture content of coal samples

After the experiment, the moisture content of coal samples at different adsorption equilibrium pressures increased to 8.0% (0.30 MPa), 8.4% (0.59 MPa), and 9.0% (0.93 MPa). The moisture content of the coal samples increased with increasing adsorption equilibrium pressure. The greater the adsorption equilibrium pressure was, the greater the pressure at the discharge end and the smaller the pressure difference between the two ends of the coal sample. According to Darcy's seepage law, the smaller the pressure difference between the two ends of the coal sample was, the smaller the water seepage velocity, resulting in a longer time for water to pass through the coal samples and a wider range of water diffusion, which increased the moisture content of the coal samples.

**Figure 5:**
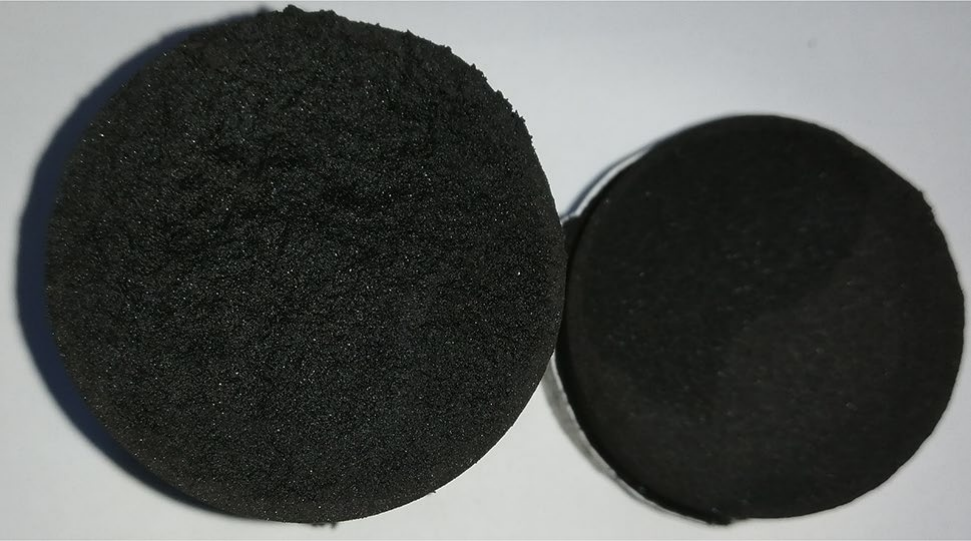
Internal moisture of coal sample after the experiment.

The water injection times of the coal samples with adsorption equilibrium pressures of 0.30 MPa, 0.59 MPa and 0.93 MPa were 10 min, 12 min and 15 min, respectively. When other experimental conditions were the same, the moisture content of the coal samples reached 11.8% after a long water injection time. Therefore, the moisture content of the coal samples under the experimental conditions in this study did not reach the saturated moisture content. This was also illustrated by the distribution of visible wetted areas inside the coal samples as shown in Fig. 5. In observing the flow behavior of water in coal samples with nuclear magnetic resonance spectroscopy (NMR), Pan et al.^35^ found that water first flowed along multiple dominant paths in the coal samples and then gradually entered other pores and fractures. The short water injection time during the experiment prevented water from fully entering all the accessible pores, making the moisture content of the experimental coal sample less than the saturated moisture content of the coal sample. The variation in the moisture content and the distribution of visible wetted areas inside the coal sample also indicated that water effectively passed through the coal sample and the water injection process was successfully simulated.

### Desorption rate

**Figure 6:**
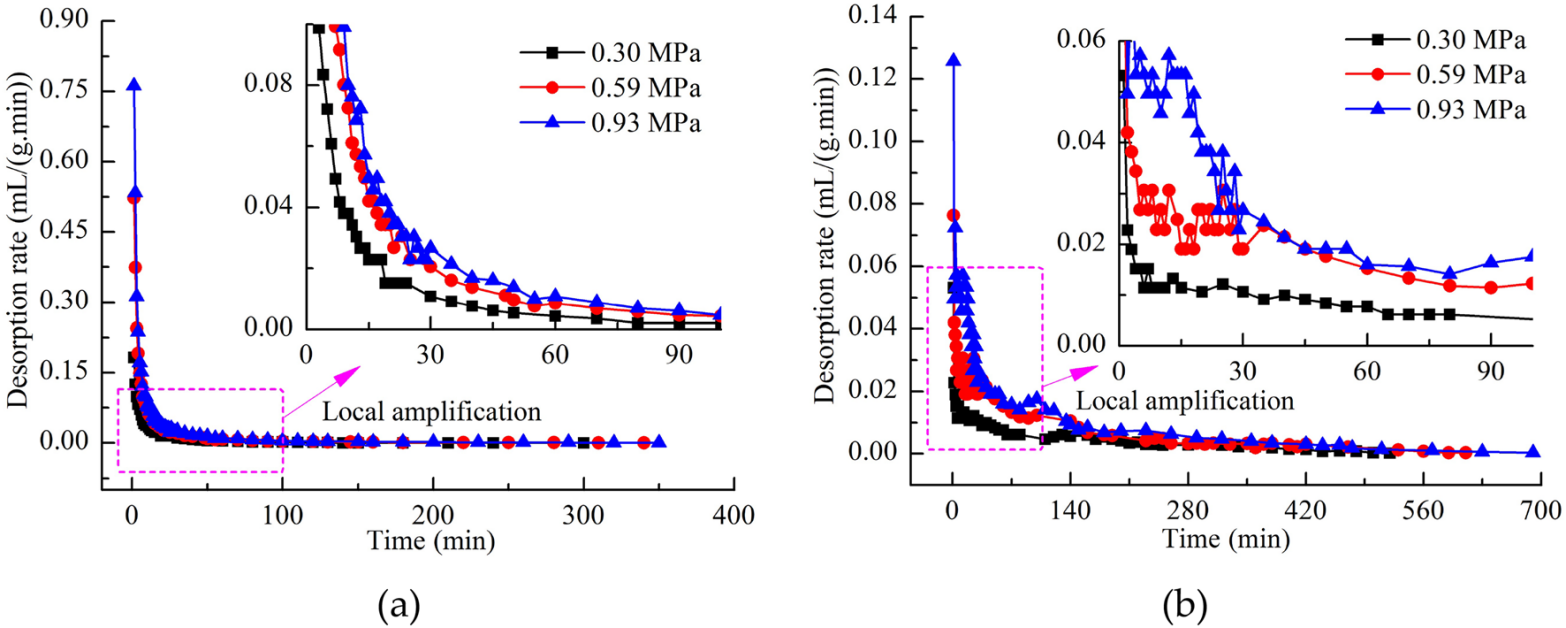
(**a**) Desorption rate of NCS. (**b**) Desorption rate of WCS.

The desorption rates of NCS and WCS increased with the adsorption equilibrium pressure, as shown in Fig. 6a and b. The desorption rate of NCS decreased continuously with time, while the desorption rate of WCS decreased in a fluctuating manner with time. For NCS, no water blocked the methane seepage channels, which caused the methane to percolate regularly along the flow channels, and the methane desorption rate continuously decayed. The presence of water in the methane seepage channels of WCS affected the flow of gas, which in turn changed the methane discharge behavior^36,37^.

**Figure 7:**
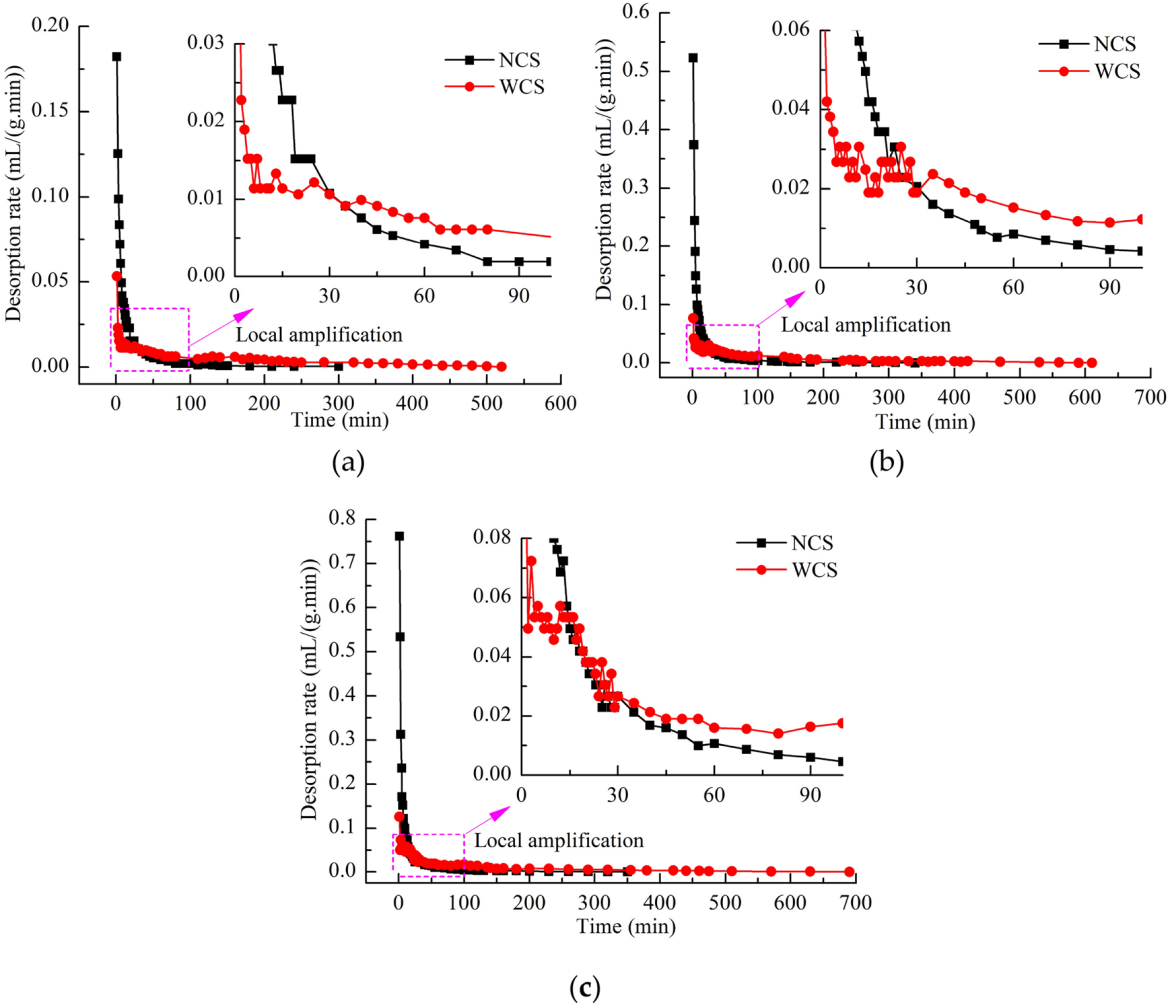
(**a**) Desorption rates of WCS and NCS at an adsorption equilibrium pressure of 0.30 MPa. (**b**) Desorption rates of WCS and NCS at an adsorption equilibrium pressure of 0.59 MPa. (**c**) Desorption rates of WCS and NCS at an adsorption equilibrium pressure of 0.93 MPa.

As shown in Fig. 7a–c, the curve of desorption rate can be divided into three stages within the desorption time. In the first stage, the desorption rate of NCS was higher than that of WCS. In the second stage, the desorption rate of NCS was approximately the same as that of WCS. In the third stage, the desorption rate of NCS was less than that of WCS. The duration of each stage was different at different adsorption equilibrium pressures. The times for the first stage were 1–30 min (0.30 MPa), 1–20 min (0.59 MPa) and 1–13 min (0.93 MPa). The times for the second stage were 1 min (0.30 MPa), 21–30 min (0.59 MPa) and 14–30 min (0.93 MPa). The third stage was from 30 min until the end of desorption. As the adsorption equilibrium pressure increased, the duration of the first stage decreased, and the duration of the second stage increased. At the same adsorption equilibrium pressure, the desorption rates of coal samples with a higher moisture content decayed more slowly. The rapid desorption rate of NCS during the early stage caused the methane content of the coal samples to decrease quickly. In contrast, the desorption rate of WCS was smaller and decayed more slowly, and water replacement of methane occurred at the same time, which caused the graphs of the desorption ratios of NCS and WCS to cross.

### Desorption amount

**Figure 8:**
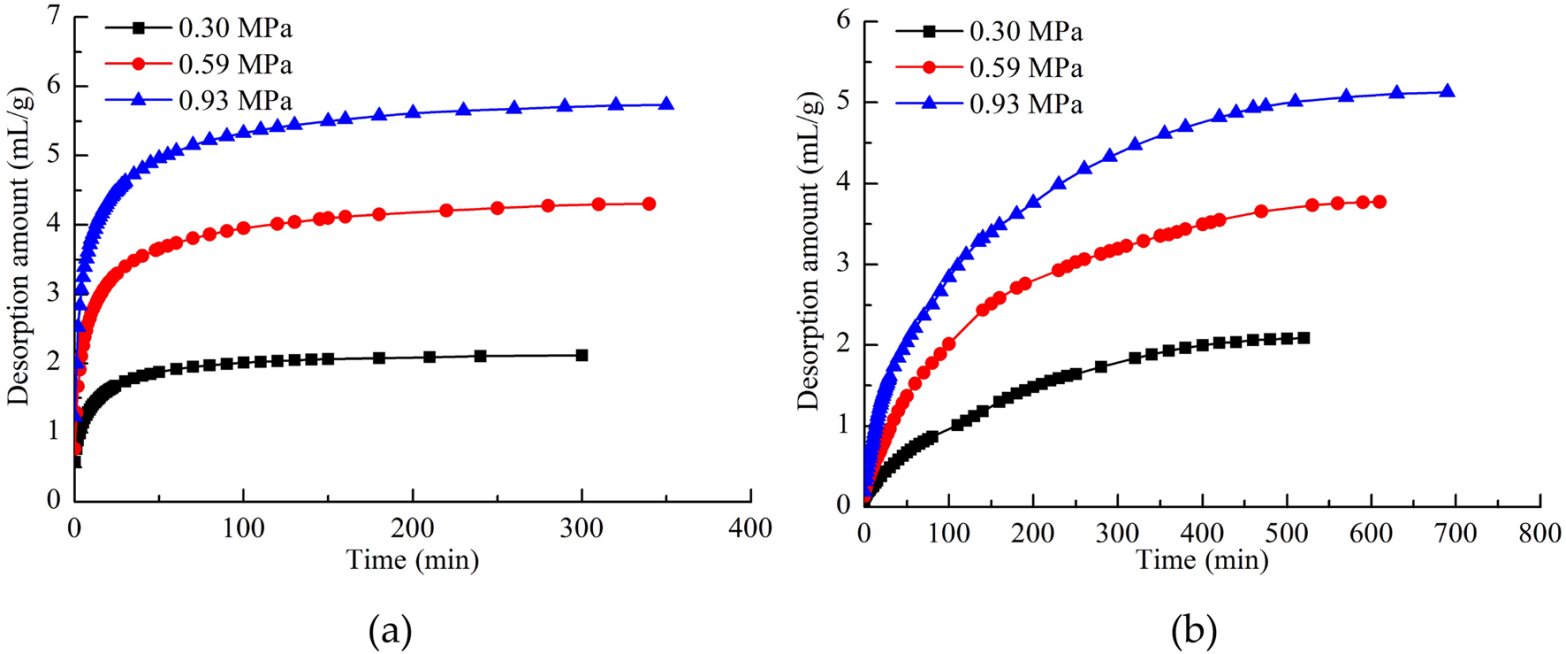
(**a**) Gas desorption amount from NCS. (**b**) Gas desorption amount from WCS.

Figure 8a and b show that the change trend in methane desorption with time from WCS was significantly different from that for NCS. The gas desorption amount of NCS increased rapidly, and it was divided into three stages: a rapid growth stage, slow growth stage, and stop stage. The methane desorption curves of WCS increased slowly, and there were only two stages: a slow growth stage and stop stage. Water makes coal samples release gas slowly, which explains the decrease in gas concentration at the working face of water-injected coal seams during mining operations^38,39^.

**Figure 9:**
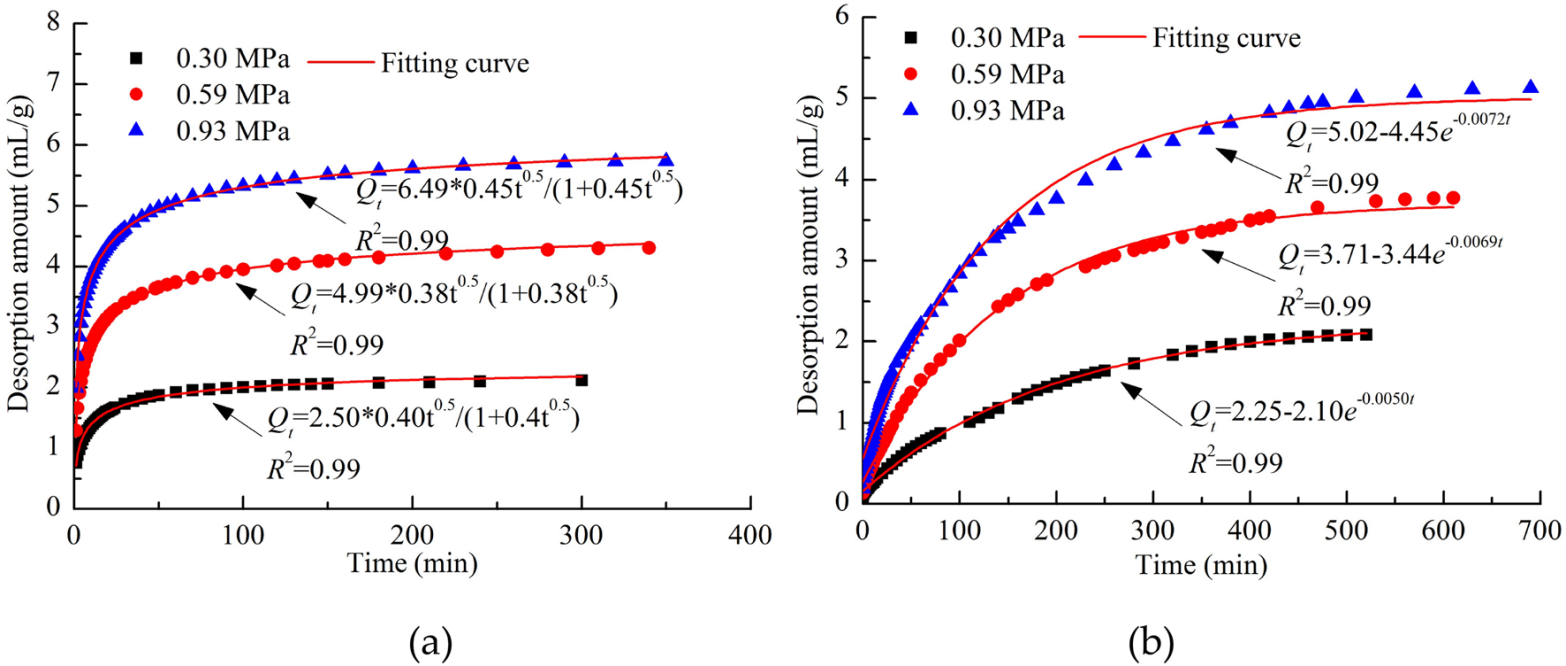
(**a**) Fitting results of NCS. (**b**) Fitting results of WCS.

The trend of coal desorption with time has many forms, such as the Barrel type^40,41^, Venter type^42^, Bolt type^43^, and Qin type^44^. According to the fitting results in this study, the change trend of the gas desorption amount of NCS conforms to the Qin type, as shown in Fig. 9a.

1 $$Q_{t} = \frac{AB\sqrt t }{{1 + B\sqrt t }},$$

where *Q*_*t*_ is the cumulative gas desorption amount at time *t* in mL/g; and *A* and *B* are desorption constants.

The methane desorption trend of WCS differed from that of NCS and conformed to the Bolt type with a goodness of fit greater than 0.99. As shown in Fig. 9b, the maximum desorption was estimated based on the fitting results.

2 $$Q_{t} = Q_{\infty } \left( {1 - Ce^{Dt} } \right),$$

where *Q*_∞_ is the maximum desorption amount of the coal sample as time approaches infinity in mL/g; and *C* and *D* are constants.

### Comprehensive influence of water injection on gas desorption behavior

The desorption ratio is defined as the ratio of the amount of methane desorbed from a coal sample to the content of methane in the coal, which is:

3 $$\eta_{d} = \frac{{Q_{t} }}{Q} \times 100\% ,$$

where ${\eta }_{d}$ is the desorption ratio of the coal sample in %; and *Q* is the methane content of the coal sample in mL.

**Figure 10:**
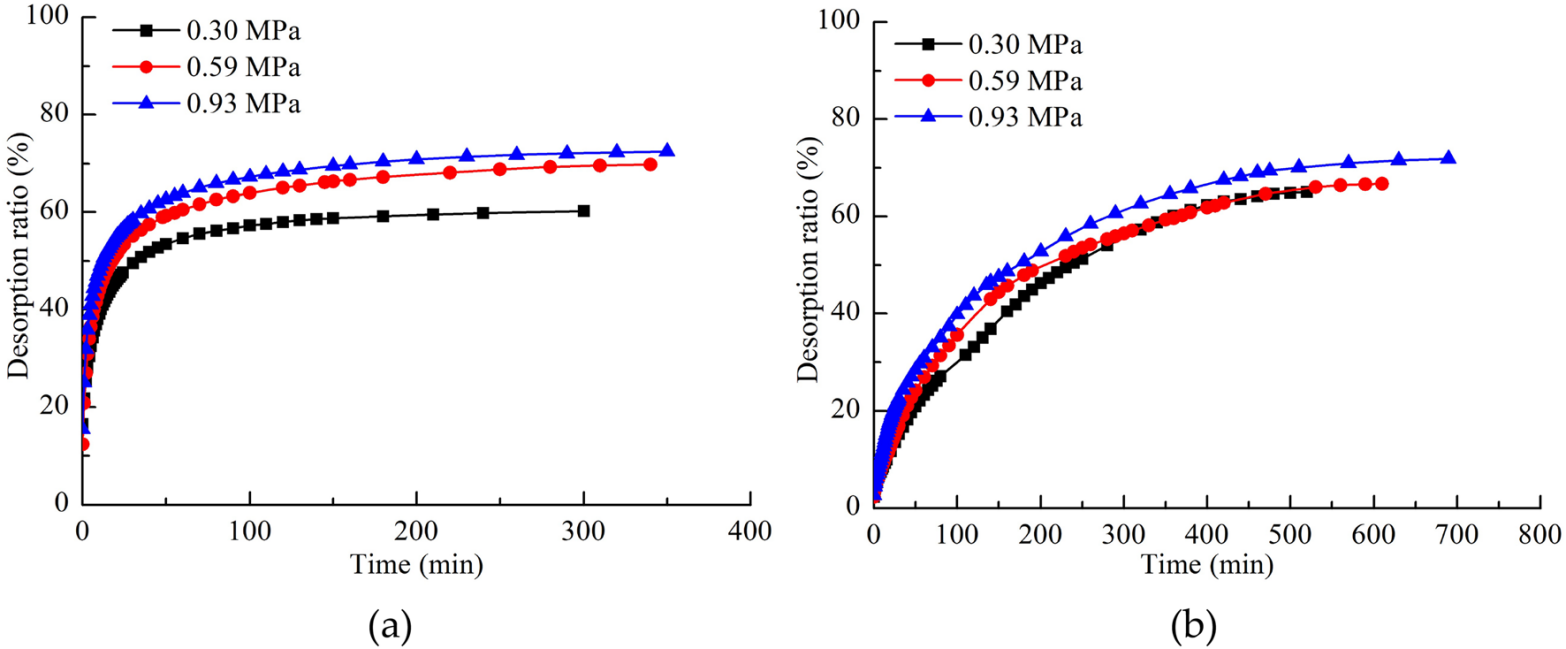
(**a**) Desorption ratio of NCS. (**b**) Desorption ratio of WCS.

As shown Fig. 10, the gas in the pipeline was considered in the calculation of the desorption ratio of NCS. However, the gas in the pipeline was not included in the calculation of the desorption ratio of WCS because this gas was driven out after water injection. As shown in Fig. 10, the desorption ratio of the coal samples followed the same trend as the desorption amount. The desorption ratio of NCS increased with the adsorption equilibrium pressure. However, the desorption ratio of WCS did not differ significantly at different adsorption equilibrium pressures, indicating that water injection caused the gas desorption amount of the coal samples to vary in a consistent manner.

The water injection discharge ratio is defined as the ratio of the sum of the displacement amount during water injection and the desorption after water injection to the methane content in coal. That is,

4 $$\eta_{w} = \frac{{Q_{w1} + Q_{w2} }}{Q} \times 100\% ,$$

where $\eta_{w}$ is the water injection discharge ratio of the WCS in %; *Q*_*w*1_ is the amount of methane displaced during water injection in mL; and *Q*_*w*2_ is the total desorption amount after water injection in mL.

**Figure 11:**
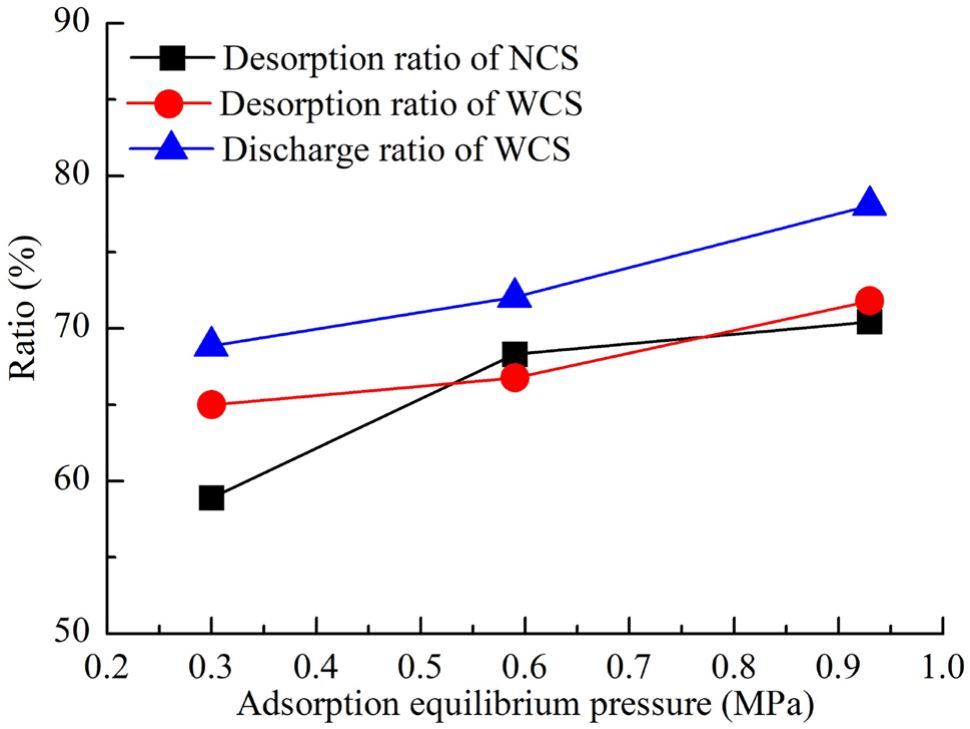
Desorption ratios and discharge ratios under different adsorption equilibrium pressures.

In Fig. 11, the desorption ratio of WCS at the adsorption equilibrium pressure of 0.59 MPa was less than that of NCS at the same adsorption equilibrium pressure, and the desorption ratios of WCS at adsorption equilibrium pressures of 0.30 MPa and 0.93 MPa were larger than those of NCS at the same adsorption equilibrium pressures, which indicated that displacement occurred during the WCS desorption process. In the desorption process, when the displacement effect was greater than the inhibition effect of water, the WCS desorption ratio was greater than the NCS desorption ratio at the same adsorption equilibrium pressure; when the displacement effect was less than the inhibition effect of water, the WCS desorption ratio was smaller than the NCS desorption ratio. At different adsorption equilibrium pressures, the NCS desorption ratio was less than the WCS water injection discharge ratio, indicating that water injection promoted the discharge of gas from coal.

After depressurization desorption, the residual methane content in NCS at different adsorption equilibrium pressures should be approximately equal, so the greater the adsorption equilibrium pressure was, the greater the desorption ratio of the coal samples, as shown in Fig. 11. According to the change in moisture content of the coal samples after the experiment, the greater the adsorption equilibrium pressure was, the greater the moisture content of the coal sample, which caused a greater adsorption equilibrium pressure, and the larger the surface of the coal sample wetted by water. Water displaced methane at the coal surface. The larger the wetted surface was, the greater the displacement amount. In addition, the greater the moisture content of the coal sample was, the greater the amount of free gas driven from the coal sample. Therefore, the WCS desorption ratio and the discharge ratio increased with the adsorption equilibrium pressure.

## Discussion

According to the above analysis, water injection reduced the gas desorption rate at the early stage of gas absorption, while water displacement improved the gas desorption rate at the late stage of gas absorption. And the replacement effect of water was greater than the inhibition effect. Therefore, water injection promoted the gas discharge.

After water is discharged from the exhaust end, the pipelines at both ends of a coal sample are filled with water. The gas discharged during the water injection process includes the gas in the pipelines at both ends of a coal sample, the free gas driven out of a coal sample, and the replacement gas. Due to the short water injection time, the amount of replacement gas is small. Without considering the amount of replacement gas, the discharge ratio of free gas is defined as the ratio of the amount of free gas driven out to the amount of free gas in a coal sample. That is

5 $$\eta_{f} = \frac{{Q_{d} }}{{Q_{f} }} \times 100\% ,$$

where *Q*_*d*_ is the amount of free gas driven out during water injection in mL; and *Q*_*f*_ is the amount of free gas in a coal sample in mL.

**Figure 12:**
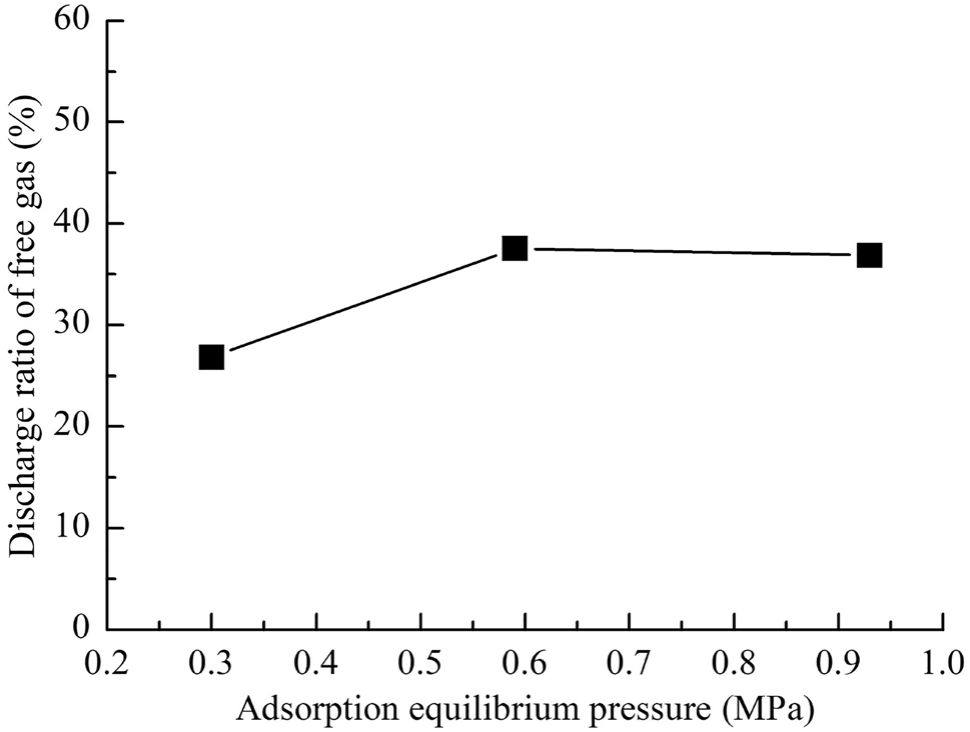
Discharge ratio of free gas under different adsorption equilibrium pressures.

As shown in Fig. 12, the discharge ratio of free gas is relatively small, indicating that water only enters a small part of the pores and fissures in the coal samples. After desorption, the moisture content of the coal sample is similar to that of the coal sample after long-term water injection, which indicates that the water at both ends of the coal sample enters more pores and fissures of the coal sample through imbibition during desorption. In the process of imbibition, water replaced the adsorbed gas and promoted the desorption of gas^22,45^. However, the gas desorption in the wet area formed during water injection was inhibited^20,24,46^. And considering the amount of free gas discharged, the total amount of gas desorption is increased by water injection. In the field, the gas emission still increased for a long time after the hydraulic measures were stopped^47–49^. This is consistent with the experimental results.

The experiment was stopped when there was water discharged from the exhaust end, resulting in a short time for water injection. The simulated conditions of the experiment accord with the conditions of the water injection area far away from the water injection borehole. In the coal seam area near the water injection borehole, the coal is immersed by dynamic water for a long time. Therefore, the gas desorption law of coal seams with long-term water injection needs to be further studied.

## Conclusions

In this study, an experimental device designed to simulate water injection were used to conduct experiments. The influence of water on gas desorption was analyzed. The conclusions are as follows.

1. The immersion of water into the pulverized coal after adsorption equilibrium leads to the phenomenon of water replacing gas, and the amount of methane displacement decreases as the diffusion of water weakens and the adsorbed gas amount in the wetted region decreases.
2. During the water injection process, the wetted area of the coal sample was relatively small. However, during the desorption process, the occurrence of capillary imbibition resulted in the water content of the coal sample approaching its saturation water content. This, in turn, increaseed the gas displacement and enhanced the gas discharge during the water imbibition process.
3. The gas desorption amount of NCS changed greatly, and the corresponding change curve can be divided into three stages: a rapid growth stage, slow growth stage, and stop stage. The amount of methane desorbed from WCS changed gently, and its change curve had only two stages: a slow growth stage and stop stage. The variation in NCS desorption amount conformed to the Qin type, and the trend for the WCS desorption amount conformed to the Bolt type.
4. The water blockage in the early stage of desorption reduced the desorption rate, while in the later stage, the replacement and imbibition effects of water increased gas emission. In combination, the NCS desorption ratio is less than the WCS water injection discharge ratio, demonstrating that dynamic water injection promotes gas desorption. Utilizing the effects of water at different stages can be employed for gas hazard control and gas extraction promotion.

## Data Availability

The data used to support the findings of this study are available from the corresponding author upon request.

## List of symbols

*Q*_*t*_: Cumulative gas desorption amount at time *t,* mL/g
*A*: Desorption constant
*B*: Desorption constant
*Q*_∞_: Maximum desorption amount of the coal sample as time approaches infinity, mL/g
C: Desorption constant
*D*: Desorption constant
$\eta_{d}$: Desorption ratio of the coal sample, %
*Q*: Methane content of the coal sample, mL
$\eta_{w}$: Water injection discharge ratio of the WCS, %
*Q*_*w*__1_: Amount of methane displaced during water injection, mL
*Q*_*w*__2_: Total desorption amount after water injection, mL
$\eta_{f}$: Discharge ratio of free gas, %
*Q*_*d*_: Amount of free gas driven out during water injection, mL
*Q*_*f*_: Amount of free gas in a coal sample, mL
